# Neuropeptide-Functionalized Gold Nanorod Enhanced Cellular Uptake and Improved In Vitro Photothermal Killing in LRP1-Positive Glioma Cells

**DOI:** 10.3390/pharmaceutics14091939

**Published:** 2022-09-13

**Authors:** Sivasoorian Siva Sankari, Ritesh Urade, Chien-Chih Chiu, Li-Fang Wang

**Affiliations:** 1 Department of Medicinal & Applied Chemistry, College of Life Sciences, Kaohsiung Medical University, Kaohsiung 807, Taiwan; 2 Department of Biological Sciences, National Sun Yat-sen University, Kaohsiung 804, Taiwan; 3 Department of Biotechnology, Kaohsiung Medical University, Kaohsiung 807, Taiwan; 4 Center for Cancer Research, Kaohsiung Medical University Hospital, Kaohsiung Medical University, Kaohsiung 807, Taiwan; 5 The Graduate Institute of Medicine, College of Medicine, Kaohsiung Medical University, Kaohsiung 807, Taiwan; 6 Department of Medical Research, Kaohsiung Medical University Hospital, Kaohsiung 807, Taiwan

**Keywords:** glioma, blood–brain barrier, neuropeptide (ANGIOPEP-2), gold nanorod, photothermal therapy, hyperthermia

## Abstract

The therapeutic modalities for glioblastoma multiforme fail badly due to the limitations of poor penetration through the blood–brain barrier and the lack of tumor targeting. In this study, we synthesized a neuropeptide (ANGIOPEP-2)-functionalized gold nanorod (GNR-ANGI-2) and systemically evaluated the cellular uptake and photothermal effects enhanced by the neuropeptide functionalization of the gold nanorod under laser or sham exposure. The expression of LRP1, the specific ligand for ANGIOPEP-2, was the highest in C6 cells among five studied glioma cell lines. The cellular internalization studies showed higher uptake of gold nanorods functionalized with ANGIOPEP-2 than of those functionalized with scrambled ANGIOPEP-2. The in vitro photothermal studies of C6 cells treated with GNR-ANGI-2 and laser showed a higher rate of apoptosis at early and late stages than cells treated with GNR-ANGI-2 without laser. Correspondingly, in vitro ROS evaluation showed a higher intensity of ROS production in cells treated with GNR-ANGI-2 under laser irradiation. The Western blotting results indicated that GNR-ANGI-2 with laser exposure activated the caspase pathway of apoptosis, and GNR-ANGI-2 with sham exposure induced autophagy in C6 cells. The current study provides in-depth knowledge on the effective time point for maximum cellular uptake of GNR-ANGI-2 to achieve a better anti-glioma effect. Moreover, by exploring the molecular mechanism of cell death with GNR-ANGI-2-mediated photothermal therapy, we could modify the nanoshuttle with multimodal targets to achieve more efficient anti-glioma therapy in the future.

## 1. Introduction

The most aggressive of all tumors, glioblastoma multiforme (GBM), has a poor prognosis and a very low survival rate. More than 150 tumors have been documented until 2020, and the tumors are mainly categorized into two groups: primary and metastatic tumors [1]. Gliomas are the most prevalent form of adult tumor, accounting for 78% of malignant brain tumors [2]. Glial tumors include astrocytomas, ependymomas, GBM, medulloblastomas, and oligodendromas [1,3]. Given the high prevalence of malignancy in adults and poor prognosis with traditional treatments, researchers struggle to develop targeted therapies. Glioblastoma shares some common properties with peripheral tumors, including elevated interstitial fluid pressure, hypoxemia, and low pH, and is unique in its tumor microenvironment [4]. Several mutations in GBM promote the survival and proliferation of glial cells in a hostile and hypoxic environment [5]. Besides the common properties of peripheral tumors, gliomas are also characterized by infiltration, making the blood–brain barrier (BBB) a significant hurdle to glioma treatment.

Receptor-mediated drug delivery is an effective strategy to overcome the BBB because brain capillary cells express several receptors, including the transferrin receptor, low-density lipoprotein-related protein 1 (LRP1), and nicotinic acetylcholine receptors [6]. Researchers have focused on developing novel dual-targeting photothermal agents (PTAs) with metallic nanoparticles and peptides/antibodies that can help to transport drugs to tumor sites crossing the BBB [7,8,9]. Peptide-based ligands are widely used to facilitate targeted drug delivery to the brain because they can be easily structured. ANGIOPEP-2, developed as a ligand for LRP1, is a 19-mer peptide derived from the human Kunitz domain [10,11].

Photothermal therapy (PTT) can be categorized as either hyperthermia (42 °C to 45 °C) or ablation therapy (>50 °C). However, ablation can cause deleterious and irreversible necrosis in cancer cells, enormous loss of normal cells, and tissue damage [12]. Regional hyperthermia has several biological effects of potential therapeutic interest [13]. Hyperthermia over 45 °C within 5–10 min can cause the necrosis of tumor cells with the irreversible denaturation of structural membrane proteins. Therefore, researchers have been paying attention to the use of PTT for glioma therapy to avoid the recurrence of GBM [1]. Several studies have been reported and are being reported on the efficient photothermal killing of gliomas using targeted PTT with varying nanoconjugates [14,15,16]. Several metal nanoparticles (NPs) used as PTAs have shown effective targeting efficiency and significant photothermal killing effects on cells [17,18,19,20]. Among metal NPs, gold NPs are the most studied material for glioma [7,21,22,23,24,25,26,27]. A review article has reported that incorporating peptide ligands or growth factors conjugated with gold NPs as PTAs further enhances the targeting efficiency of the glioma [28]. For example, Lee et al. reported that rabies-virus-coated gold nanorods not only bypassed the BBB but also responded to near-infrared laser (808 nm) irradiation, emitted heat, and effectively suppressed brain tumors [27]. However, few studies reported on the mechanism of glioma cell death using those PTT agents.

**Scheme 1:**
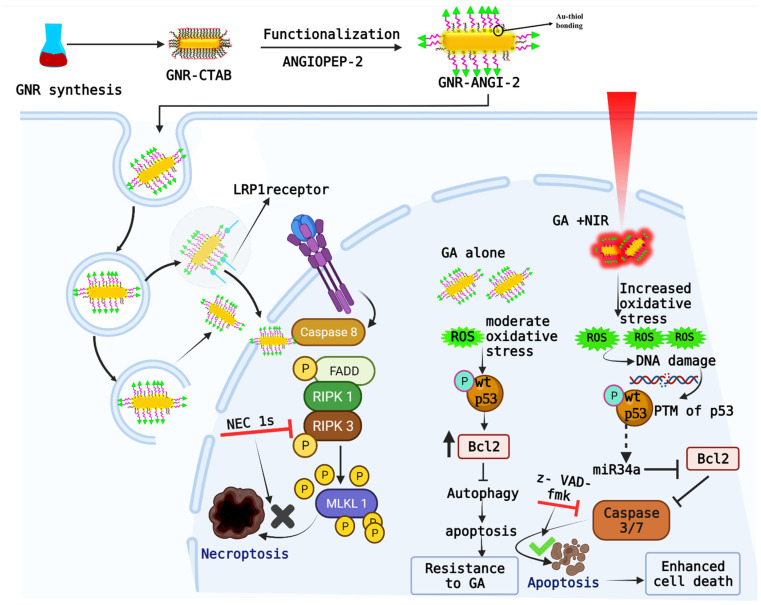
Construction of tailor-made GNR-ANGI-2 (GA) and its internalization through LRP1 receptor-mediated endocytosis into glioma cells and the possible mechanism involving cell death with and without light irradiation. The blood–brain barrier and intracellular pathway layout for endocytosis were created with BioRender.

To fill the research gap and develop an efficient PTT agent, the current study explored the molecular mechanism of cell death in C6 cells with PTT using a PTA (GNR-ANGI-2) under laser or sham exposure. We developed near-infrared (NIR)-responsive gold nanorods (GNRs) in the first biological window (λ = 650–950 nm), with deep penetration and significant photo-damage. Further, we functionalized their surface with ANGIOPEP-2 (GNR-ANGI-2) as a nanoscaled shuttle to transport GNRs across the BBB and achieve targeted PTT in the brain. The objective of the study was to develop a neuropeptide-conjugated photothermal agent that could cross the BBB for efficient PTT in glioma. Beyond the role in PTT, we studied the cell-killing mechanism induced by GNR-ANGI-2 under laser and sham exposure in C6 cells as shown in Scheme 1.

## 2. Materials and Methods

### 2.1. Reagents and Antibodies

MTT reagent was purchased from Sigma-Aldrich (St. Louis, MO, USA); we also acquired DMSO (Sigma Aldrich), LRP1 antibody (Genetex; GTX79841; 1:1000 dilution; GeneTex, Irvine, CA, USA), Calcein acetoxymethyl ester and an Annexin V/FITC-propidium iodide (PI) staining kit (Invitrogen Molecular Probes, Eugene, OR, USA), Propidium iodide (PI; 1:1000; Sigma-Aldrich).

### 2.2. Cell Culture

ALTS1C1 cells (a murine astrocytoma cell line) were obtained with the courtesy of Dr. Hsin-Cheng Chiu, Department of Biomedical Engineering and Environmental Sciences at National Tsing Hua University, Taiwan. F98, 9L, and C6 cells (originally purchased from ATCC^®^, Manassas, VA, USA) were obtained from Dr. Ren-Jei Chung, Department of Chemical Engineering and Biotechnology at National Taipei University of Technology, Taiwan. U87 cells were originally purchased from Bioresource Collection and Research Centre (BCRC; Hsinchu City, Taiwan). These glioma cells were cultivated and maintained at 37 °C under humidified 5% CO_2_ in DMEM, 10% heat-inactivated fetal bovine serum (FBS), 2 mM L-glutamine, and 100 μg/mL penicillin–streptomycin, changed twice weekly.

### 2.3. LRP1 Expression on Glioma Cells

To select glioma cells with the highest LRP1 expression, 1 × 10^6^ glioma cells/mL were harvested and suspended in cold FACS buffer (10% FBS in PBS). For blocking, 100 μL of cell suspension was incubated with 3% BSA/PBS for 1 h at room temperature (RT). The cell suspension was pelleted using centrifugation at 400 g for 5 min at 4 °C. The diluted LRP1 antibody (Genetex; GTX79841) (1:1000 dilution in 3% BSA/PBS, 10 μg/mL) was added to cells and incubated for 1 h at RT. Cells were washed three times using centrifugation at 400× *g* for 5 min. After the last centrifugation, the supernatant was removed, and the pellet was resuspended with 1 mL of 3% BSA/PBS. The cell suspension was immediately analyzed using a flow cytometer (Beckman Coulter Inc., Bera, CA, USA).

### 2.4. Cellular Uptake of GNR-ANGI-2 in C6 Cell Line

To determine the uptake of conjugated nanorods into glioma cells, an atomic adsorption spectroscopy (AAS) analysis was performed. C6 cells were seeded in 12-well plates at a density of 1 × 10^5^ cells/well and incubated for 24 h to achieve 90% confluency. The culture medium was removed, and cells were treated with GNR-ANGI-2 (20 μg/mL Au ion concentration) for corresponding time points (0.5, 2, 4, 6, 8, and 12 h). After treatment with the material, cells were collected from the culture dish with a prewarmed (37 °C) 0.25% trypsin solution at specific time intervals. The cell suspension was counted with a hemocytometer and then transferred to a 15 mL tube to measure the absorbance of the gold atom using AAS. A total of 2 × 10^4^ cells from each sample were analyzed for Au content. First, a five-point standard curve was plotted with 1000 ppm of standard gold colloid diluted to a maximum of 100 ppb to check the Au content of the samples. The collected cell suspension was dissolved in aqua regia for the digestion of gold. A volume of 2 mL of sample was loaded into the graphite furnace for each analysis. Three replicates were taken for each sample analyzed. The concentration (mg/L) and percentage of the internalized gold ions into the cells was quantified using AAS.

### 2.5. Cytotoxicity Assay of GNRs and Conjugates

Photothermal cytotoxicity was studied against C6 cells. C6 cells were seeded in 96-well plates at a cell density of 5 × 10^3^ cells/well. After an incubation period of 24 h, samples (20 µg/mL Au ion concentration) were added and incubated for 2 h. After incubation, the medium was discarded to remove the unbound nanoconjugates and replaced with fresh medium. C6 cells treated with the free medium served as controls. To evaluate the photothermal killing effect, C6 cells were irradiated with an 808 nm NIR laser (1 W cm^−2^) for 24 min.

### 2.6. Live and Dead Assay

Cells were seeded in 12-well plates at an initial density of 1 × 10^5^ cells/well. Cells were irradiated with an 808 nm laser with an optimized power source in the absence and presence of 20 µg/mL GNR-ANGI-2 for 24 min. As a control, cells were incubated in a culture medium alone or with 20 µg/mL GNRs. After photothermal treatment, cell survival was visualized using a calcein-AM/PI double staining assay (Invitrogen, Molecular Probes). For this purpose, cells were washed with PBS buffer solution. Then, 1 mL of 3 µM calcein-AM was added to each well and incubated at 37 °C for 20 min, followed by incubation with 1 mL of 2.5 µM PI for 5 min at RT, and washed with PBS for 15 min at 37 °C. Fluorescence images were acquired with an inverted Leica DMIRB microscope furnished with a digital camera (Leica DC100, Wentzler, Germany).

### 2.7. Evaluation of ROS Production

The levels of intracellular ROS were measured using a 2′,7′-dichlorofluorescin diacetate (DCFDA) dye. DCFDA is a cell-permeable fluorophore that cellular esterases deacetylate to a nonfluorescent compound oxidized by the ROS in cells into 2’,7′-dichlorofluorescin (DCF). Fluorescent DCF was analyzed with a fluorescent spectrophotometer at excitation/emission of 485 nm/535 nm. C6 glioma cells were cultured onto 24-well culture plates for 24 h. On the following day, cells were treated with GNR-ANGI-2 (20 μg/mL Au ion concentration) with or without N-acetyl cysteine (NAC) and incubated in a 5% CO_2_ incubator at 37 °C overnight. NAC is an effective antioxidant and was used as an ROS inhibitor to test whether DCF fluorescence was produced in response to ROS production. Media containing test materials were replaced with fresh media. Cells were exposed to sham or laser at 1 W cm^−2^ for 3 min. Following light irradiation and 24-h incubation, cells were rinsed thrice and pelletized using centrifugation at 4000 rpm for 5 min. Cells were redispersed in PBS buffer. The cell suspension was incubated with 10 µM DCFDA at 37 °C for 45 min. Untreated cells and cells treated with H_2_O_2_ (50 µM) with or without NAC were set as negative and positive controls, accordingly. The fluorescence intensity of DCF was measured at 535 nm using a flow cytometer (Beckman Coulter FC 500) at an excitation wavelength of 488 nm.

### 2.8. Apoptosis Assay

C6 cells were seeded at 2 × 10^5^ cells/ well in a 6-well plate and incubated overnight. Cells were then incubated with GNR-ANGI-2 (with sham or laser exposure) for 24 h at 37 °C to analyze the stages of apoptosis using an Annexin V-FITC apoptosis detection kit. After treatment, both adherent and suspended cells were collected, washed with PBS, and resuspended in 100 μL of binding buffer. Afterward, 5 μL of FITC Annexin V and 10 μL of PI were added to the cell suspensions and incubated in the dark (20 min at RT). Subsequently, 400 μL of binding buffer was added to the cells and was analyzed using flow cytometry. Apoptotic stages were resolved with the rate of apoptotic and necrotic cells. Labeling patterns indicated different cell populations, with FITC- and PI-negative cells representing viable cells; FITC-positive and PI-negative, early apoptotic cells; FITC and PI-positive, late apoptotic cells; and FITC-negative and PI-positive, necrotic cells. The fluorescence intensity of Annexin-V-FITC was measured at 530 nm at an excitation wavelength of 488 nm using an FL1 detector and PI using an FL2 detector. An average of 10,000 events were collected for each sample.

### 2.9. Evaluation of Cell-Death Pattern via MTT Assay

Glioma cells in the log phase (4 × 10^4^ cells/well) were seeded in a 96-well plate. After monolayer formation, cells were treated with GNR-ANGI-2 (20 µg/mL Au ion concentration). Cells alone were used as control group. It has been reported that the RIPK1–RIPK3–MLKL pathway is critical for the induction of necroptosis [29]. We studied the cell-death pattern by blocking the apoptotic and necrotic pathways using the inhibitors necrostatin (Nec-1) and carbobenzoxy-valyl-alanyl-aspartyl-[*O*-methyl]-fluoromethyl ketone (Z-VAD) [30]. To test whether necroptosis or apoptosis was involved in cell killing, we pretreated glioma cells with RIPK1 inhibitor (Nec-1) or pan-caspase inhibitor (Z-VAD) for 4 h and then treated with GNR-ANGI-2 for 12 h. Z-VAD or Nec-1 was added at a final concentration of 20 µM in their respective wells. Afterwards, 808 nm NIR laser exposure was applied at the above-indicated conditions. Following 24-h incubation of cells, MTT reagent was added and incubated for 4 h for transformation by mitochondrial reductase. Sequentially, the resultant formazan crystals were dissolved with DMSO and analyzed using spectrophotometry at 490 nm (Tecan Microplate Reader).

### 2.10. Evaluating the Integrity of Lysosomal Membrane

Briefly, C6 cells were seeded onto a 6-well plate for 24 h and incubated for 12 h with GNR-ANGI-2 (20 µg/mL Au ion concentration) after monolayer formation. Cells were treated under laser or sham exposure at 808 nm, 1 W cm^−2^, for 24 min. Afterward, cells were washed twice with PBS, redispersed in complete medium and stained with 5 μg/mL acridine orange (AO) at 37 °C for 30 min. After further rinsing with PBS, wells were replenished with new medium and observed under the fluorescence microscope. AO was excited at 488 nm, and emission signals were detected at 530 nm (green color) and 590 nm (red color) (Leica DMI6000B fluorescence microscope, Wentzler, Germany).

### 2.11. Western Blotting Analysis

To find the cell-death pattern caused by PTT, the protein expression of the cells after material treatment with laser or sham exposure was studied using Western blotting to confirm the upregulation and downregulation of proteins. Briefly, glioma cells in the log phase (1 × 10^6^ cells/dish) were seeded in a 60 mm dish overnight. On the next day, cells were treated with GNR-ANGI-2 at a 20 µg/mL concentration with laser or sham exposure. Cells alone were used as the control group. After material or laser treatment and post incubation, cell lysates were extracted from C6 cells. The lysates were then rinsed twice with 1X PBS and redispersed in RIPA buffer (71009; Merck, Darmstadt, Germany). Protein expression was detected using a BCA assay kit (Bio-Rad Laboratories, Hercules, CA, USA). Equal amounts of protein samples were separated and transferred to PVDF (polyvinylidene difluoride) membranes using SDS-PAGE. The membrane was then blocked for 3 h at 37 °C with 5% non-fat milk powder in Tris-buffered saline with 0.1% Tween 20 (TBST). The blocked PVDF membrane was then incubated with primary antibodies including p53 (1:5000; catalog No. 10442-1-AP; Proteintech), Bcl-2 (1:000; catalog No. 12789-1-AP; Proteintech), caspase-3 (1:1000; catalog No. NB100-56708; Novus biotech), caspase-7 (1:1000; catalog No. NB100-56529; Novus biotech), LC-3 B (1:1000; catalog No. 2775s; Cell Signaling Technology), and GAPDH (1:10,000; catalog No. AF-7021; Affinity Biotech) overnight at 4 °C. On the next day, the PVDF membrane was washed thrice with TBST and then incubated with the secondary antibodies specific to the primary antibodies for one hour at RT. Subsequently, the PVDF membrane was washed three times with TBST, and the immunoreactive blot signals were detected using enhanced chemiluminescence (ECL) reagent with a western blotting analyzer (Amersham Imager 680, GE Healthcare and Bioscience ab, Princeton, NJ, USA).

### 2.12. Statistical Analysis

Data were from three individual experiments and were presented as means ± SEs. Student’s t test was used to analyze the statistical significance of each group. The statistical significance was assessed using GraphPad Prism 9 software (GraphPad, San Diego, CA, USA). In all statistical analyses, differences with *p* values < 0.05 were considered significant.

## 3. Results and Discussion

### 3.1. Synthesis and Characterization of GNR-ANGI-2 and GNR-SC-ANGI-2

**Figure 1:**
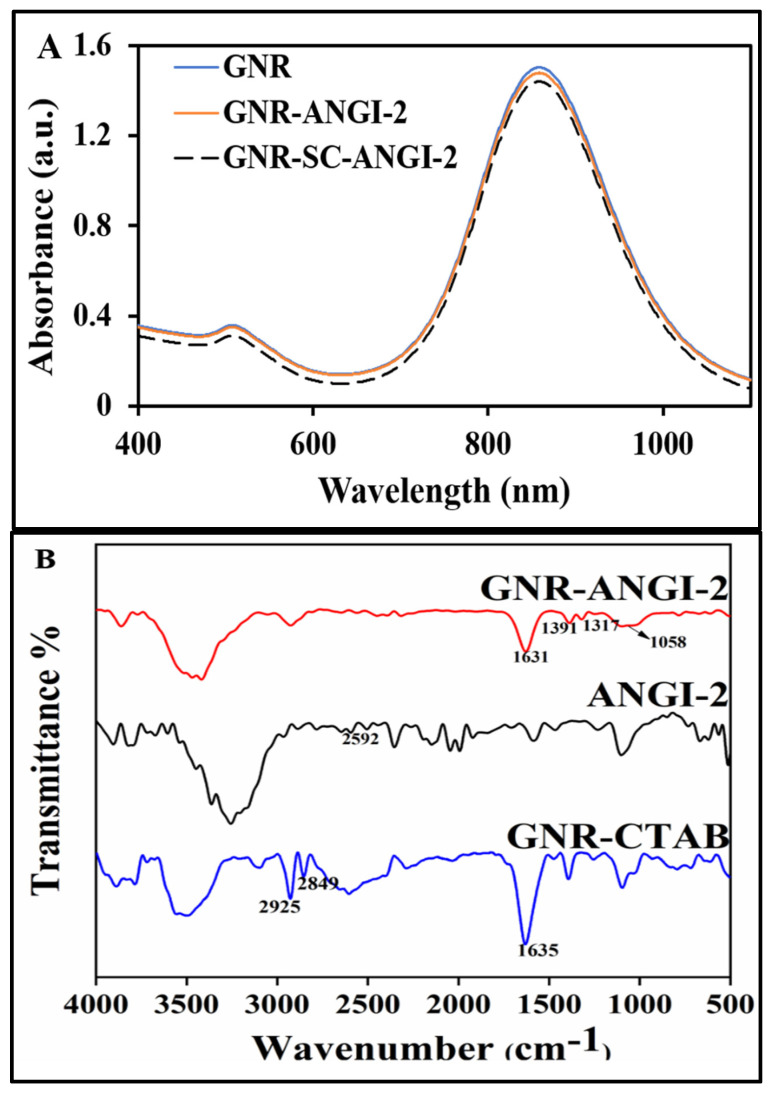
Characterization of GNR-CTAB and GNR-peptide conjugates. (**A**) UV–Vis absorbance spectra of GNR-CTAB, GNR-ANGI-2, and GNR-SC-ANGI-2. (**B**) FTIR spectra of GNR-CTAB, peptide, and GNR-peptide conjugate.

The synthesis of GNR-ANGI-2 was performed as described in the previous publication [31]. Briefly, to synthesize the GNR-peptide conjugate, we first synthesized GNRs and then conjugated them with neuropeptide modified at the C-terminus with cysteine ANGI-2 (GNR-ANGI-2) and scrambled ANGI-2 (GNR-SC-ANGI-2). The size and aspect ratio details of GNRs gained from TEM images were shown in our previous article [31]. The zeta potentials of GNRs, GNR-SC-ANGI-2, and GNR-ANGI-2 were 44.6, 19.7, and 11.45 eV, respectively, presenting a strong positive zeta potential for GNRs, a weak positive one for GNR-SC-ANGI-2, and a weaker positive one for GNR-ANGI-2. The conjugation efficiency of GNR-ANGI-2 was higher than that of GNR-SC-ANGI-2, owing to a higher removal of CTAB from the GNR surface, leading to a weaker positive zeta potential (Figure S1A). The conjugation efficiencies of ANGI-2 onto GNR were 65, 53, 33, and 5%, corresponding to 20, 15, 10, and 5 μM infeed. The concentration of peptide conjugated to GNRs was quantified using a standard curve of an ANGI-2 peptide at 275 nm. The peptide conjugation efficiency appeared to increase with the increase in peptide concentration (Figure S1B). The UV–Vis spectra in Figure 1A show that the GNRs before and after peptide conjugation had a longitudinal surface plasmon band centered at 859 nm, indicating the good stability of the GNRs after peptide conjugation. The slight decrease in absorbance intensity could be due to the successful conjugation of the peptide with GNRs, which led to variations in the refractive index and the oscillation of electrons on the gold nanorod surface. As shown in Figure 1B, the strong FTIR absorption bands at 2925 and 2849 cm^−^^1^ in GNR-CTAB were attributed to the C-H stretching vibration of methyl and methylene groups relative to the hydrocarbon tail of CTAB. The stretching vibration of hydroxyl groups that appeared at 3455 cm^−1^ in GNR-CTAB and GNR-ANGI-2 might have been due to the fact that the samples were dispersed in an aqueous solution and cast onto the ATR-FTIR plate. The representative band of free thiol in the 2500–2600 cm^−1^ region of the peptide spectra (ANGI-2) disappeared in GNR-ANGI-2 due to conjugation with Au.

### 3.2. Photothermal Effects upon Laser Irradiation

**Figure 2:**
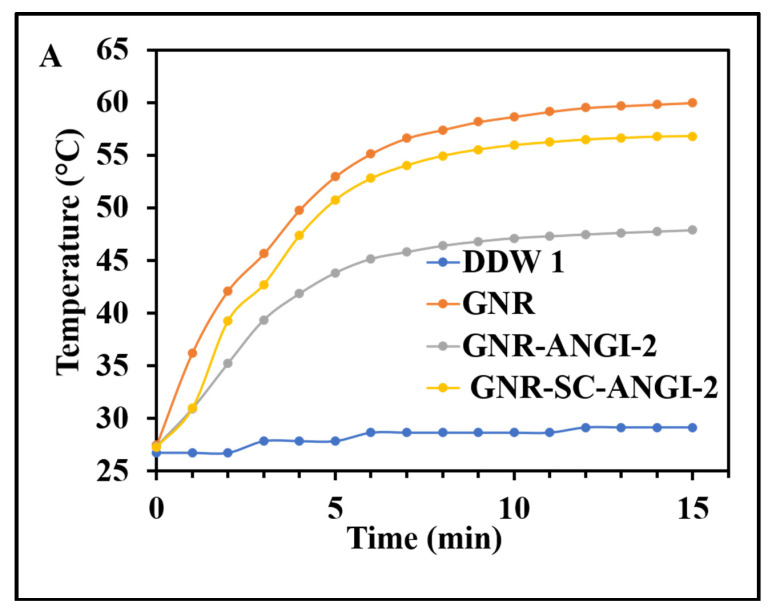

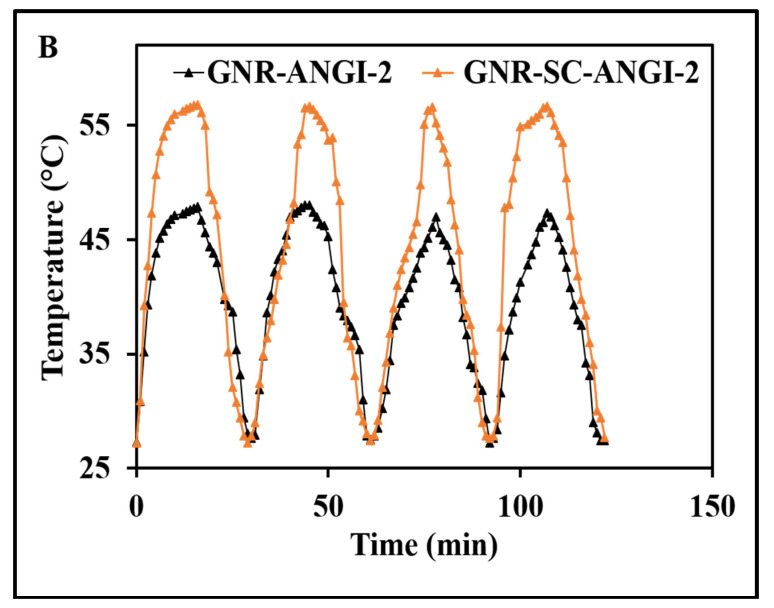
Temperature elevation profile of GNR-CTAB and GNR-peptide conjugates. (**A**) Temperature elevation of DDW, GNRs, GNR-ANGI-2, and GNR-SC-ANGI-2 conjugates at 100 μg/mL Au ion concentration with 1 W cm^−2^ in deionized water. (**B**) Reusability of GNR-ANGI-2 and GNR-SC-ANGI-2 evaluated by subjecting the GNR-peptide conjugates at 100 µg/mL Au ion concentration to 4 cycles of temperature rising and lowering under 808 nm laser irradiation (on) for 15 min, followed by sham exposure for 15 min (off) in each cycle.

Because of the localized plasmon resonance of GNRs, they can be used in laser-induced PTT. To evaluate the capacity of the ANGI-2-functionalized gold nanorod as a photothermal agent in PTT, the temperature raise tendency was established under laser exposure. The power density of the 808 nm laser at 1 W cm^−2^ was selected as suitable from the results of our previous study [31]. The photothermal conversion performance of GNRs, GNR-ANGI-2, and GNR-SC-ANGI-2 in DDW was evaluated upon laser irradiation for 15 min. As shown in Figure 2A, with GNR-ANGI-2, the temperature smoothly increased and reached a maximum value of 45 °C after 6 min. With GNRs or GNR-SC-ANGI-2, on the other hand, the temperature rapidly rose and reached a plateau of 45 °C within 3 and 3.5 min, respectively. The difference in thermal conversion efficiency between GNR-SC-ANGI-2 and GNR-ANGI-2 could be attributed to the efficiency of peptide conjugation onto GNRs. The higher amount of ANGI-2 covered on the surface of GNRs caused interfacial thermal resistance. The thermal stability and reusability of GNR-ANGI-2 and GNR-SC-ANGI-2 were evaluated with four consecutions of irradiation (on/off) with an 808 nm NIR laser. The GNR suspension with 100 μg/mL Au ions, was treated with laser for 15 min and then cooled to RT for 15 min without laser (off). As shown in Figure 2B, there was a trivial temperature gradient between adjoining peaks in each cycle, indicating that the GNR-peptide conjugates were stable for up to four cycles of NIR laser irradiation.

### 3.3. LRP1 Expression of Cell Lines

**Figure 3:**
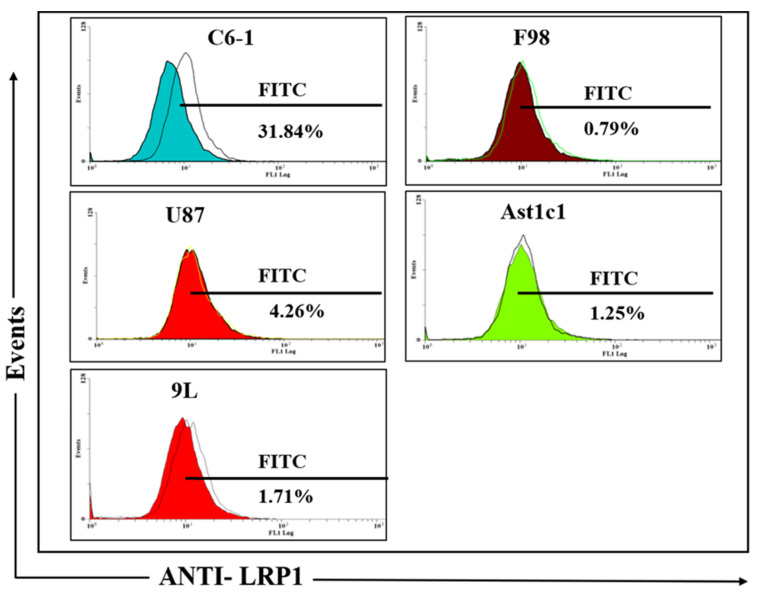
LRP1 expression studies on glioma cell lines. Histogram overlays of the corresponding cell populations (LRP1-null cells and anti-LRP1-antibody-labeled cells). Left, control LRP1-null cells (shaded) and anti-LRP1-antibody-labeled cells (lines). The histogram shows the percentage of FITC fluorescence. Flow cytometry quantified the values on histogram markers to represent the percent of cells positive for FITC fluorescence (FL1).

The LRP1 expression of cells is a key factor for selective targeting, as glioma cells overexpress LRP1 on their surfaces [32]. To select a cell line with the highest LRP1 expression, the LRP1 expression of five different glioma cell lines was studied using a conjugated primary antibody (FITC-conjugated LRP1 antibody). The study groups contained null cells without antibodies and cells treated with FITC-conjugated LRP1 antibodies. As shown in Figure 3, among the five cell lines tested, C6 (31.84%) cells showed the highest LRP1 expression compared with the other cell lines; even though U87 (4.26%), 9L (1.71%), Ast1c1 (1.25%), and F98 (0.79%) cells had LRP1 expression, it was not significant in comparison with that of C6 cells. Thus, the C6 cell line was chosen as the model cell line for further testing.

### 3.4. Cell Viability

Cell viability was studied with GNR-ANGI-2 against NIH3T3 cells with and without irradiation [33]. To rule out the effect of the laser, a control group without materials was exposed to the laser, and cell viability was not affected, indicating that NIH 3T3 cells were not sensitive to laser irradiation under the experimental conditions. GNR-ANGI-2 under sham or laser exposure maintained up to 75–60% of cell viability at the maximum concentration of material tested (200 µg/mL). GNR-ANGI-2 with or without laser treatment showed cell viability of approximately 80% up to an Au ion concentration of 100 µg/mL (Figure S2).

### 3.5. Cellular Internalization of Gold-Peptide Conjugate

**Figure 4:**
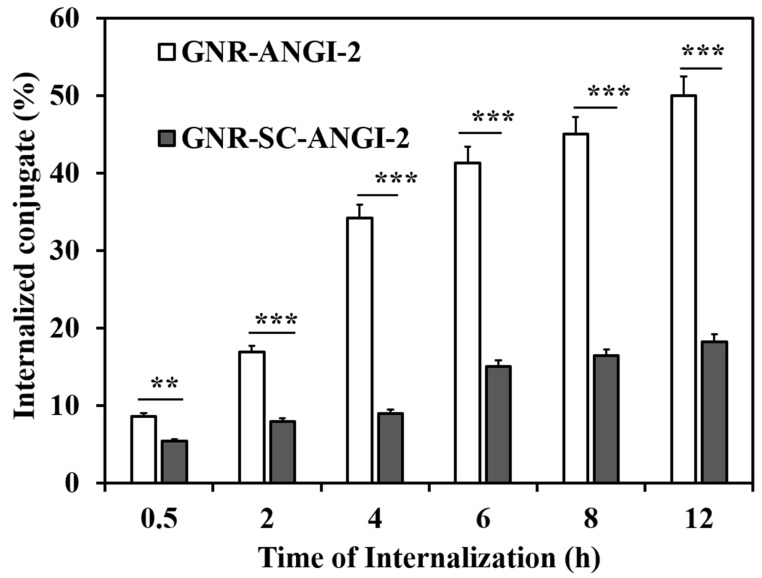
Time-dependent cellular internalization experiment of GNR-ANGI-2 and GNR-SC-ANGI-2. Following incubation of C6 cells with GNR-ANGI-2 and GNR-SC-ANGI-2 at a concentration of 20 µg/mL, the internalization rate was calculated for varying time points (including 0.5, 2, 4, 6, 8, and 12 h) through AAS analyses. (*n* = 3; ** *p* < 0.01 and *** *p* <0.001).

The uptake of GNR-ANGI-2 into cancer cells is essential for noninvasive therapy and has negligible toxicity to healthy cells. To examine the effect of incubation time on the enhancement of cellular uptake, the cellular internalization of Au ions at different time points (0.5, 2, 4, 6, 8, and 12 h) was analyzed in C6 cells using AAS. The total mass of associated Au ions per sample was determined with three independent measurements (n = 3) and presented as the percentage of internalization (Figure 4). The measured amount of Au ions proved the C6 cell internalization of GNR-ANGI-2. Accordingly, the cellular uptake of the GNR-ANGI-2 conjugate was enhanced with increased incubation time and reached 50% after 12 h. With GNR-SC-ANGI-2, the cellular uptake was ~18.25% after 12 h. This result indicates that the rearrangement of the amino-acid sequence in ANGI-2 had a strong impact on the cellular uptake of the nanoparticle. Moreover, the cellular uptake of GNR-ANGI-2 was highly significant compared with that of GNR-SC-ANGI-2 at all the given time points.

### 3.6. In Vitro Therapeutic Effect of GNR-ANGI-2 against Glioma Cells

**Figure 5:**
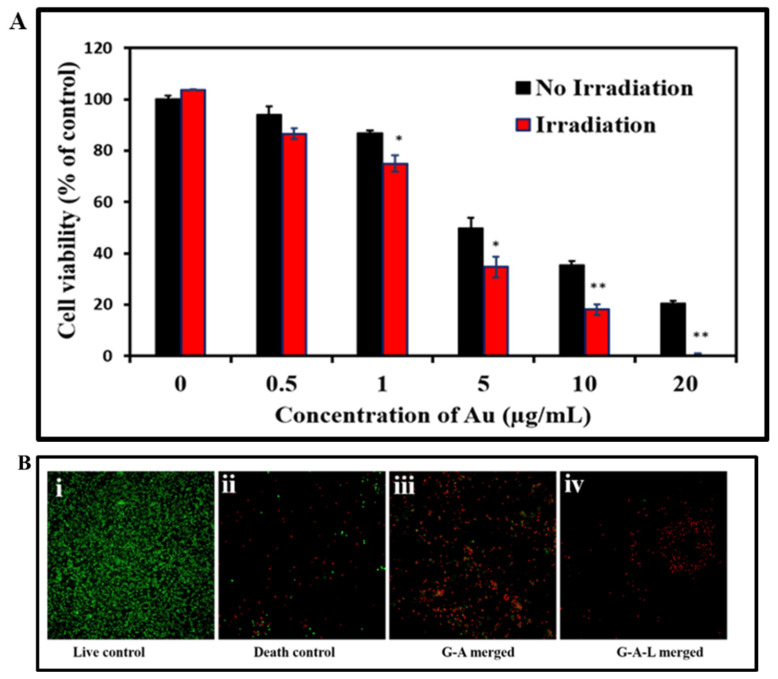
In vitro therapeutic effect of GNR-ANGI-2 against glioma cells. (**A**) Cytotoxicity evaluation of GNR-ANGI-2 against C6 glioma cells. Viability assay after 24 h incubation at 37 °C with subsequent NIR laser exposure of 808 nm at 1 W cm^−2^ for 24 min. The results represent the means and standard deviations (SDs) of three independent experiments (* *p* ≤ 0.05, ** *p* ≤ 0.01). (**B**) C6 cells stained with calcein-AM and propidium iodide to visualize live (green) and dead (red) cells: (**B**(**i**)) cells alone; (**B**(**ii**)) cells treated with alcohol; (**B**(**iii**)) cells treated with GNR-ANGI-2 (20 μg/mL Au ion concentration) for 12 h; (**B**(**iv**)) cells treated with GNR-ANGI-2 (20 μg/mL Au ion concentration) exposed to laser for 24 min and incubated for 12 h.

The cytotoxicity of GNR-ANGI-2 to the growth of C6 cells was evaluated using an MTT assay (Figure 5A). It was found that at 0.5 µg/mL (Au ion concentration), GNR-ANGI-2 (L+) and (L−) showed very low cytotoxicity. Cell viability remained at 90% and 95%. At 1 µg/mL, GNR-ANGI-2(L+) and (L−) still had little effect on cell viability. At 5 µg/mL, the cell viability of GNR-ANGI-2 (L−) and (L+) reduced to 55% and 38%. At 10 µg/mL, the GNR-ANGI-2 (L−) and (L+) showed more cytotoxicity, and cell viability was reduced to 35% and 18%. Finally, when the cells were treated with GNR-ANGI-2 (L−) and (L+) at 20 µg/mL, cell viability further dropped to 20% and 0%. The above results demonstrated that material with and without irradiation had significant cytotoxicity toward glioma cells at a maximum concentration (20 µg/mL). The IC 50 value was calculated as 3.3 ± 0.05 µg/mL under light exposure. It is a very mature achievement that gold nanorods modified with targeting units and used in combination with NIR exposure may impart high toxicity to cancer cells and negligible effects to normal cells [34,35,36,37]. The analogous result was found here, i.e., GNR-ANGI-2 showed negligible toxicity to normal cells (NIH3T3 cells) but killed cancer cells (C6 cells). This result may be attributed to the higher LRP1 expression in C6 cells, which increased the effective cellular uptake of GNR-ANGI-2, resulting in a higher cell-killing effect.

Cell viability after hyperthermia treatment was evaluated with calcein AM-PI staining. No significant cell death was detected in untreated cells, and few live cells were observed in the alcohol-treated death control cells (Figure 5B(i,ii)). Without laser treatment, GNR-ANGI-2 significantly affected cell viability, as indicated by the increase in cells stained with PI (Figure 5B(iii)). However, the cells treated with GNR-ANGI-2 and laser irradiation caused 100% cell death, as determined by the absence of calcein-positive cells in the experimental conditions, whereas all C6 cells present in the culture were PI-positive (Figure 5B(iv)). The cell loss was found in GNR-ANGI-2 after laser treatment due to the severely damaged cells, which were removed during staining and media transfer; the same phenomenon was observed in the case of the death controls treated with alcohol.

### 3.7. Evaluation of ROS

**Figure 6:**
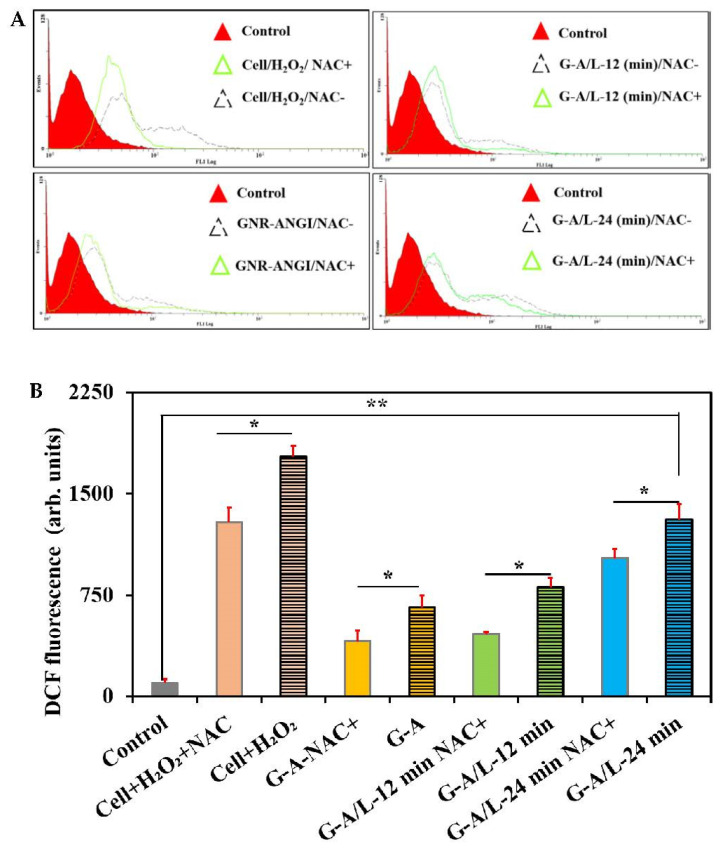
Evaluation of Reactive Oxygen Species (ROS). The fluorescence density of dichlorofluorescin (DCF) was measured via flow cytometry after C6 cells were treated with GNR-ANGI-2 with or without N-acetyl cysteine (NAC) at a concentration of 5 mM under laser or sham exposure with two irradiation time points (12 min or 24 min) and stained with the DCFDA reagent for 20 min. (**A**) Representative histogram of C6 cells with different treatments using flow cytometry. The log fluorescence (X-axis) illustrates the DCF intensity of fluorescence for the number of cells counted. (**B**) The bar graph represents quantified data of fluorescence intensity of DCF, representing a remarkable increase in ROS in G-A/L-24 min. The values are presented as means ± SEMs. (*n* = 3; * *p* <0.05 and ** *p* <0.01).

ROS generation associated with GNR-ANGI-2 upon laser exposure was examined using a DCFDA assay. NIR laser stimuli can induce cellular damage via the hyperthermia effect and heat-stress-induced ROS production during PTT to target cancer cells. N-acetyl cysteine (NAC) is an inhibitor of ROS. The intracellular ROS generation in C6 cells treated without test material or treated with H_2_O_2_, H_2_O_2_ /NAC, GNR-ANGI-2, GNR-ANGI-2-L+ (808 nm, 1W cm^−2^, 12 min), or GNR-ANGI-2-L+ (808 nm, 1W cm^−2^, 24 min), was confirmed using DCF fluorescence intensity using flow cytometry. Figure 6A,B show the representative histogram of DCF fluorescence intensity and its graphical representation of C6 cells exposed to different treatments. DCF fluorescence intensity was directly proportional to the amount of ROS produced in the cells. As shown in Figure 6B, cells treated with test material and without laser exposure exhibited a lesser amount of ROS production. In contrast, cells treated with test material and with laser exposure exhibited notable ROS production. The increase in the time of laser exposure influenced ROS production. The fluorescence intensity in GNR-ANGI-2–24 with 24 min laser irradiation had a strong shift compared with GNR-ANGI-2-12 with 12 min laser irradiation. We clarified that the above-mentioned cell damage was mainly caused by ROS induced with the laser exposure of GNR-ANGI-2 using NAC as an ROS inhibitor. The expression of ROS was significantly reduced in NAC-treated cells with sham or laser exposure. The positive control treated with H_2_O_2_ showed the highest yield of ROS. ROS production was significantly dependent on the duration of laser exposure. ROS is associated with apoptosis activation and is considered an important regulator of apoptosis [38].

### 3.8. Assessment of Cell-Death Pattern

#### 3.8.1. Annexin V-FITC/PI Dual Staining Assay

**Figure 7:**
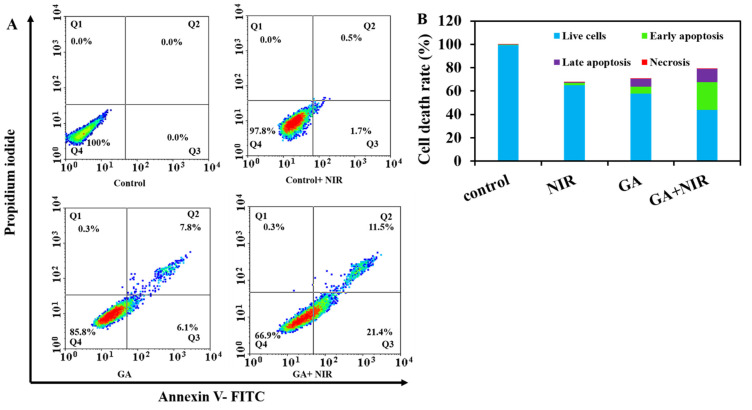
Apoptosis and necrosis of C6 cells analysis using flow cytometry with annexin V-FITC/propidium iodide (PI) dual staining. (**A**) Two-dimensional scatter plots of annexin V-FITC vs. PI from flow cytometry. Cells staining negative for annexin V-FITC and PI in the lower left quadrant are live cells. Cells staining positive for annexin V-FITC and negative for PI in the lower right quadrant represent early apoptosis. Cells staining positive for annexin V-FITC and PI in the upper right quadrant are late apoptotic. (**B**) Summary of the annexin V-FITC apoptosis assay results showing the percentages of cell-death modes: necrosis, late apoptosis, early apoptosis, and live cells.

Apoptosis was evaluated with a flow cytometer via Annexin V-FITC and PI labeling, and the rate of apoptotic cells was expressed in percentage. The scatter plot in Figure 7 indicates that cells in the Q1, Q2, Q3, and Q4 quadrants were necrotic, late apoptotic, early apoptotic, and live cells, respectively. As exposed in Figure 7A, the control group without any treatment showed an insignificant number of cells in the early and late apoptotic stages with 100% of live cells. A slight elevation in cells treated with laser alone resulted in an insignificant increase in the apoptotic cell rates of Q3 (1.7%) and Q2 (0.5%), respectively. When C6 cells were exposed to GNR-ANGI-2, a considerable number of cells in Q3 (6.1%) and Q2 (7.8%) were mainly in the apoptosis phase. A further increase in cells treated with GNR-ANGI-2 under laser exposure showed a significant increase in the apoptotic cell rates of Q3 (21.4%) and Q2 (11.5%), respectively.

#### 3.8.2. MTT Assay

**Figure 8:**
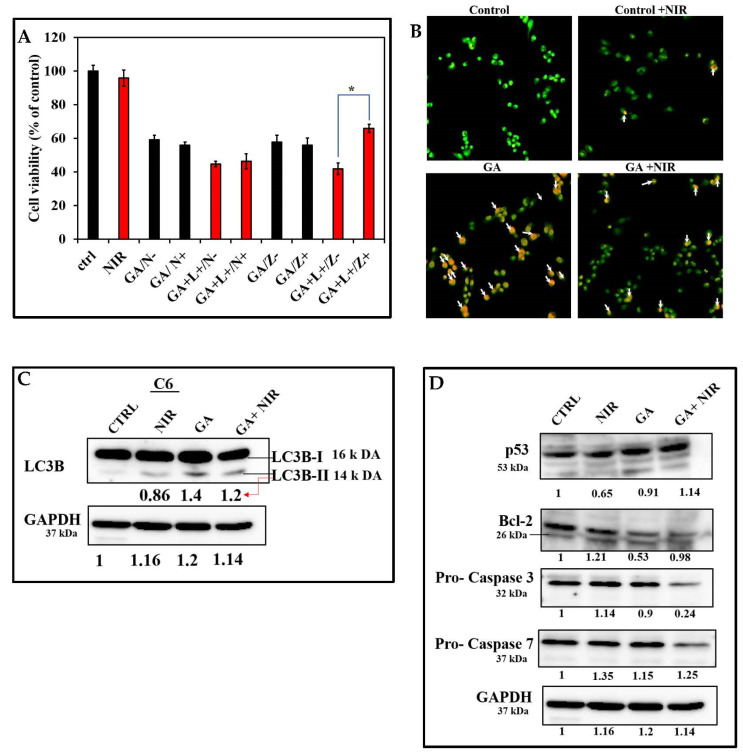
Cell-death patterns of C6 cells treated with GNR-ANGI-2 under laser or sham exposure. (**A**) Cell cytotoxicity pattern of C6 cells preincubated with caspase (Z-VAD) inhibitor or RIPK1 (Nec-1) inhibitor for 4 h and irradiated with laser (12 min); cell viability was evaluated using the MTT assay; cells treated with media were used as control. The bar graph represents the percentages of cell viability respective to the treatment conditions. Error bars represent the standard deviations of 3 experiments (*n* = 3, * *p* < 0.05). (**B**) C6 cells incubated with medium (control), laser (control+ NIR), GNR-ANGI-2 (GA), and GR-ANGI-2 (GA+NIR) were treated with 5 µg/mL AO for 30 min; then, images were recorded under fluorescence microscopy. Red fluorescence (white arrows) indicates acid vesicular organelles stained with acridine orange within the cells. (**C**) LC3-II processing in C6 cells treated with GNR-ANGI-2 under laser or sham exposure, and control and control treated with laser incubated for 24 h using Western blotting. (**D**) Changes in protein expression in C6 cells treated with GNR-ANGI-2 under sham or laser exposure. Representative Western blot results show the expression of p53, caspase-3, caspase-7, and Bcl-2 proteins. ImageJ was used to analyze the fold change in protein expression.

Photothermal treatment can produce hyperthermia in tumor sites, killing tumor cells through necrosis or apoptosis induction [39]. Previous studies have reported that PTT could cause cell death by either apoptosis or necroptosis depending on the hyperthermia and induced stress [40]. We investigated whether the cells treated with GNR-ANGI-2 could induce necrotic or apoptotic cell death. It has been stated that the RIPK1–RIPK3–MLKL pathway is pivotal for the induction of necroptosis and that the caspase pathway is critical for the induction of apoptosis. To test whether cells treated with GNR-ANGI-2 were involved in tumor killing by necroptosis or apoptosis mediated by PTT, C6 cells were treated with GNR-ANGI-2 L+/L− or PBS in the presence of necrostatin-1 (Nec-1), an RIPK1 inhibitor that blocks the necroptosis pathway [30]. We also used the pan-caspase inhibitor, carbobenzoxy-valyl-alanyl-aspartyl-[*O*-methyl]-fluoromethyl ketone (Z-VAD), to block the apoptotic pathway [41]. After treatment with the 808 nm NIR laser, cell viability was determined using the MTT assay [40]. As displayed in Figure 8A, the percentage of cell viability in the cells treated with GNR-ANGI-2 and laser irradiation without Z-VAD was 41.7% and increased to 65.9% with previous treatment with Z-VAD. In the cells treated with GNR-ANGI-2 without laser irradiation, cell viability remained almost similar with or without Z-VAD treatment from 56% to 59%. After blocking necroptosis with the addition of Nec-1, the decrease in cell viability in GNR-ANGI-2-treated cells under laser or sham exposure was not significant (46.3% and 44.7%). In the cells treated with GNR-ANGI-2 in the presence or absence of Nec-1, the viability remained the same (55.8% and 59.1%). The above result implies that GNR-ANG-2 with laser exposure seemed to kill tumor cells mainly by apoptosis regulated by the caspase signaling pathway. A proposed mechanism of PTT killing tumor cells is illustrated in Scheme 1.

#### 3.8.3. Lysosome Integrity Assessment

The lysosomal membrane permeation of cells exposed to GNR-ANGI-2 with laser or sham exposure was analyzed using AO staining. C6 cells were treated with GNR-ANGI-2 under laser or sham exposure and stained with AO staining medium for 30 min. The fluorescence microscopic images are shown in Figure 8B; the orange, fluorescent color representing positive cells stained by acidic vesicular organelles (AVOs) increased in the cells treated with GNR-ANGI-2 under laser or sham exposure. Still, with GNR-ANGI-2 under sham exposure, the number of AVOs was significantly higher compared with GNR-ANGI-2 under the laser. Cells alone with or without laser irradiation showed green fluorescence, indicating insignificant formation of AVOs. This result implied that the cells treated with GNR-ANGI-2 induced autophagic initiation. To further demonstrate that GNR-ANGI-2 indeed triggered the autophagy mechanism in C6 cells, the processing marker of LC3-I to LC3-II was detected using Western blotting. As shown in Figure 8C, the processing of LC3-II was detected in both GNR-ANGI-2 under sham or laser exposure. GNR-ANGI-2 showed a high processing of LC3-II, and GNR-ANGI-2 with laser exposure showed moderately significant processing. These results suggested that GNR-ANGI-2 could induce autophagy in C6 cells.

#### 3.8.4. Western Blotting Assay

To examine the apoptotic signaling pathway involved in C6 cell death, the protein levels of p53, pro-caspase-3/7, and Bcl-2 were quantified and shown in Figure 8D. With GNR-ANGI-2 under laser exposure, the Bcl-2 and pro-caspase-3/7 protein levels decreased, and the p53 protein levels increased significantly compared with the control and GNR-ANGI-2 without laser irradiation. The possible cell signaling pathway with GNR-ANGI-2 under laser exposure may be as follows: GNR-ANGI-2 with laser exposure produced significant ROS as evaluated with the DCFDA assay. Hence, the increased oxidative stress induces the post-translational modification of p53, which activates miR34; activated miR34a causes the depression of Bcl-2; Bcl-2 then activates caspase-3/7, thus inducing apoptosis and enhanced cell death as proposed in Scheme 1. A similar study with Fe_3_O_4_@Au composite magnetic NPs modified with cetuximab under laser exposure has shown intrinsic apoptosis with the upregulation of caspase-3/8 and -9 in human glioma cells (U251) [23]. In contrast, cells treated with GNR-ANGI-2 alone showed moderate ROS production and an insignificant change in Bcl-2 expression fold as compared with the control group. This result may imply that GNR-ANGI-2, due to moderate oxidative stress, induces p53 to upregulate Bcl-2 expression [42], which antagonizes autophagy initiation, eventually leading to less significant cytotoxicity or resistance. Numerous studies in preclinical models have shown that the inhibition of pro-survival autophagy kills tumor cells by promoting apoptotic cell death [43]. The modulation of the autophagic response is also considered a promising strategy to improve cancer therapy in clinical trials [44].

## 4. Conclusions

We developed a neuropeptide-conjugated gold nanorod, GNR-ANGI-2, to enhance cellular uptake and achieve efficient, targeted photothermal therapy in gliomas. For comparative purposes, we synthesized GNR-SC-ANGI-2 with scrambled ANGIOPEP-2. GNR-ANGI-2 showed enhanced and efficient cellular uptake in C6 cells as compared with GNR-SC-ANGI-2. The internalization amount of GNR-ANGI-2 increased with time. Cellular uptake was indeed performed through LRP1-mediated transcytosis. The cytotoxicity studies showed that C6 cells exposed to GNR-ANGI-2 with or without laser exposure had severe cytotoxicity. The evaluation of intracellular ROS production in C6 cells showed that GNR-ANGI-2 with laser significantly increased ROS production and enhanced the photothermal killing efficiency against C6 cells. Moreover, provided with the ROS results, the killing mechanism involved might be ROS-mediated apoptosis. To further elucidate the cell-death pattern, pan-caspase and RIPK1 inhibitors were used to inhibit the pathways and study the inhibitors’ effect on the viability of C6 cells. The Nec-1 inhibitor-treated cell viability studies showed that necroptosis was not the mechanism of killing with GNR-ANGI-2 under sham or laser exposure. To confirm the mechanism of killing, the results of Western blotting confirmed the promotion of autophagy, which is completely related to stress intensity and plays a dual role in carcinogenesis as a promoter or suppressor. The cell-killing mechanism involved in cells treated with GNR-ANGI-2 under sham exposure might have been due to autophagy-mediated pathways; however, with GNR-ANGI-2 under laser exposure, the possible mechanism of cell death might have been due to the caspase-induced apoptosis pathway. The cell-death pathway of GNR-ANGI-2 depends on exposure to sham or laser; thus, this material can be tuned for efficient cancer killing using different pathways.

## Data Availability

Not applicable.

## Supplementary Materials

The following supporting information can be downloaded at: https://www.mdpi.com/article/10.3390/pharmaceutics14091939/s1, Figure S1: Zeta potential of GNRs, GNR-SC-ANGI-2, and GNR-ANGI-2 and peptide concentration plot on the GNR-ANGI-2 conjugate versus peptide infeed concentration; Figure S2: Evaluation of cell viability in NIH3T3 cells; Figure S3: Whole blot of p53; Figure S4: Whole blot of Bcl-2; Figure S5: Whole blot of Caspase-3; Figure S6: Whole blot of Caspase-7; Figure S7: Whole blot of LC3B-I & II; Figure S8: Whole blot of GAPDH. The Supporting Information also contains a brief description of materials, GNR synthesis, GNR–peptide conjugate synthesis, characterization, temperature elevation profile, and cell viability studies. References [31,45] are cited in the supplementary materials.
