# Supplementary material for: Neuropeptide-Functionalized Gold Nanorod Enhanced Cellular Uptake and Improved In Vitro Photothermal Killing in LRP1-Positive Glioma Cells
Source: Pharmaceutics. 2022 Sep 13;14(9):1939. doi: 10.3390/pharmaceutics14091939 (PMC9504705; doi:10.3390/pharmaceutics14091939)
Supplement: Supplementary file 1 [file pharmaceutics-14-01939-s001.zip › pharmaceutics-1859088-si.pdf]

Supporting information:

Neuropeptide-Functionalized Gold Nanorod Enhanced Cellular Uptake and Improved In Vitro Photothermal Killing in LRP1-Positive Glioma Cells

Sivasoorian Siva Sankari [1], Ritesh Urade [2], Chien-Chih Chiu [2,3,4,5,6,*] and Li-Fang Wang [1,6,*]

[1] Department of Medicinal & Applied Chemistry, College of Life Sciences, Kaohsiung Medical University, Kaohsiung 807, Taiwan

[2] Department of Biological Sciences, National Sun Yat-Sen University, Kaohsiung 804, Taiwan

[3] Department of Biotechnology, Kaohsiung Medical University, Kaohsiung 807, Taiwan

[4] Center for Cancer Research, Kaohsiung Medical University Hospital, Kaohsiung Medical University, Kaohsiung 807, Taiwan

[5] The Graduate Institute of Medicine, College of Medicine, Kaohsiung Medical University, Kaohsiung 807, Taiwan

[6] Department of Medical Research, Kaohsiung Medical University Hospital, Kaohsiung 807, Taiwan

* Correspondence: cchiu@kmu.edu.tw (C.-C.C.); lfwang@kmu.edu.tw (L.-F.W.)

1. Materials and Methods

1.1 Materials:

Hydrogen tetrachloroaurate (III) hydrate (HAuCl4. 3H2O, Alfa Aesar) 99.99%, Hexadecyltrimethylammonium bromide (CTAB, Acros, 99 + %), L-Ascorbic acid (AA, Everdine technology, Mw 176.12; 99%), Silver nitrate (AgNO3, Alfa Aesar, Mw 169.87; 99.9+%), sodium borohydride (NaBH4, Sigma-Aldrich, Mw: 37.83; 99%), Neuropeptide (ANGIOPEP-2), Scrambled Angiopeptide (Sc-ANGIOPEP-2) modified at the C-terminal with cysteine amino acid, Gendanio, Mw: 2404.65, Working solutions were prepared in Double Distilled Water (ddH2O).

1.2 GNRs synthesis:

CTAB-stabilized gold nanorods were synthesized as described previously [1]. Precursor solution of same composition (5 mL of 5 mM $HAuCl_4$ with 5 mmol CTAB and bringing the volume to 50 mL with ddH_2O) prepared in two individual tubes, poured into one flask and bring to a final volume of 100 mL. Under vigorous stirring, 120 µL of 100 mM $AgNO_3$ solution was added dropwise. Within 5 min, 600 µL of 100 mM ascorbic acid (AA) and 40 µL of 1.6 mM $NaBH_4$ solution was added simultaneously, and the mixture was left under stirring for 150 min. The obtained solution was centrifuged at 10000 rpm for 15 min. The supernatant was discarded, and the pellet was repeatedly washed with ddH_2O under sonication, followed by centrifugation under the same conditions. Finally, the GNRs were dispersed in ddH_2O.

1.3 GNR-peptide conjugate synthesis:

Metal peptide conjugates prepared by partial ligand exchange with the thiol groups of the peptide sequence ANGIOPEP-2 (TFFYGGSRGKRNNFKTEEYC) modified with a cysteine end. The surface CTAB of GNR replaced with the peptides can also reduce the cytotoxicity of the functionalized GNRs. Following purification, GNRs were redispersed in ddH_2O and then subjected to conjugation with peptide (ANGIOPEP-2) with the Au ion concentration at 20 µg mL^{-1}. The mixture was left on a sonicator for over eight hours to obtain homogeneous GNR-ANGI-2. The resulting mixture was collected and purified twice with centrifugation at 6000 rpm for 10 min and dispersed in ddH_2O.

1.4 Characterization:

UV/Vis Absorption spectra of all the synthesized nanostructures were recorded on the UV-Vis-NIR region using a UV-vis-NIR spectrophotometer (JASCO, V-670). The morphology and size of the synthesized nanomaterial were observed with Transmission Electron Microscopy (TEM) (Hitachi H-

7500) and High-Resolution Transmission Electron Microscopy (HRTEM) (JEOL JEM-2100F electron microscope). Zeta potentials were measured using a Zeta potential and particle size measuring instrument (Otsuka ELSZ-2000 instrument). Gold ion concentration and elemental compositions of samples were analyzed using an Atomic Adsorption Spectroscopy (AAS).

1.5 Temperature elevation profile

A 200 μL volume of GNR with an Au ion concentration of 100 ppm was added to each well of a 96-well microtiter plate. The photoinduced temperature change of the solution was achieved using a CW NIR diode laser of 808 nm at 1 W cm^{-2} for 3 min [2]. The temperature change was captured with an infrared camera, and the digital values were recorded with the help of a thermocouple combined with a digital thermometer (DER EE DE-3003 K-type).

2 Cell studies

2.1 Cell viability assay

The cell viability was studied using an MTT assay. For the viability assay, cells were seeded into 96-well plates (5×10^3 cells/well, eight replicates for each condition). After 24 h, glioma cells were treated with GNR-ANGI-2 ranging from 0 to 20 μg/mL for 24 h. After incubation, the cells were treated with sham or laser exposure. The cultures were washed twice in phosphate-buffered saline to remove any residual GNRs, and the tetrazolium dye, 3-(4,5-dimethilthiazol-2)-2,5-diphenyl-2H-tetrazolium bromide (MTT, 5 mg/mL in phosphate-buffered saline), was added to the medium for 4 h. After removal of the medium, the precipitated formazan crystals were dissolved in optical grade dimethyl sulfoxide (100 μL). The absorbance of each well is measured spectrophotometrically at 570 nm. Cell viability is estimated using equation (1) and expressed as cell viability percentages.

Cell viability (%) = $OD_{570(test)}/OD_{570(control)}$ --------------------- (S1)

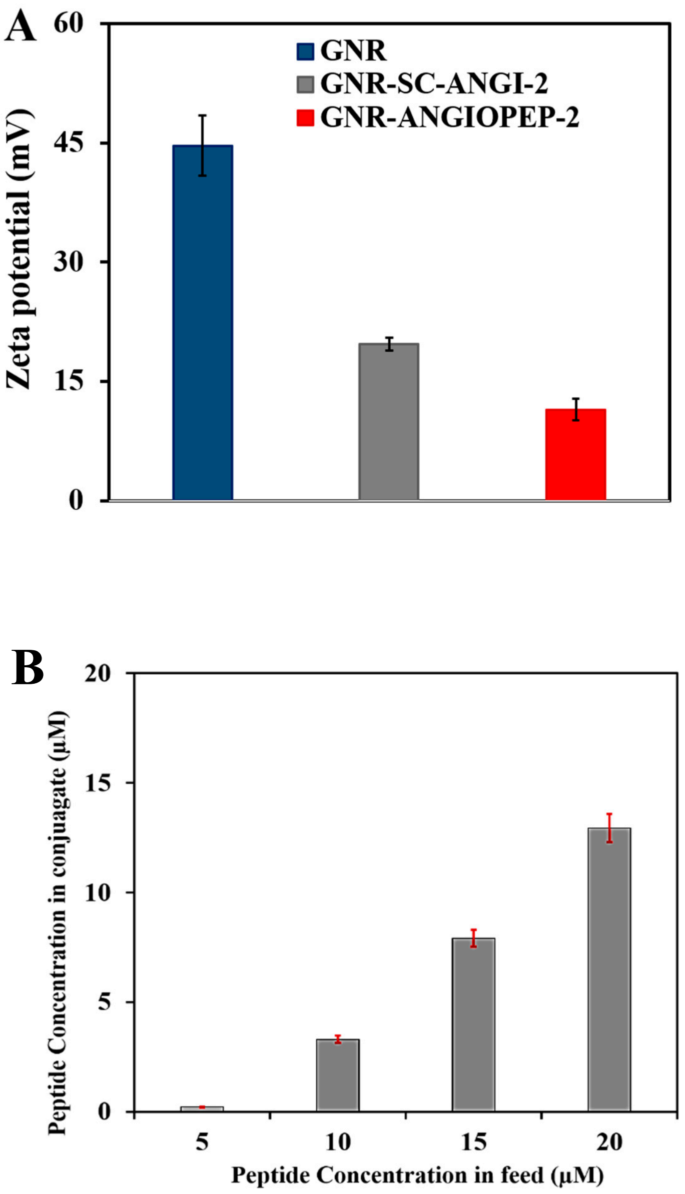

Figure S1. (**A**) Zeta potential of GNRs, GNR-SC-ANGI-2, and GNR-ANGI-2. (**B**) Peptide concentration plot on the GNR-ANGI-2 conjugate versus peptide infeed concentration, with fixed GNR (Au ion concentration 20 μg/mL) and different concentrations of peptide infeed studied with UV-vis spectroscopy.

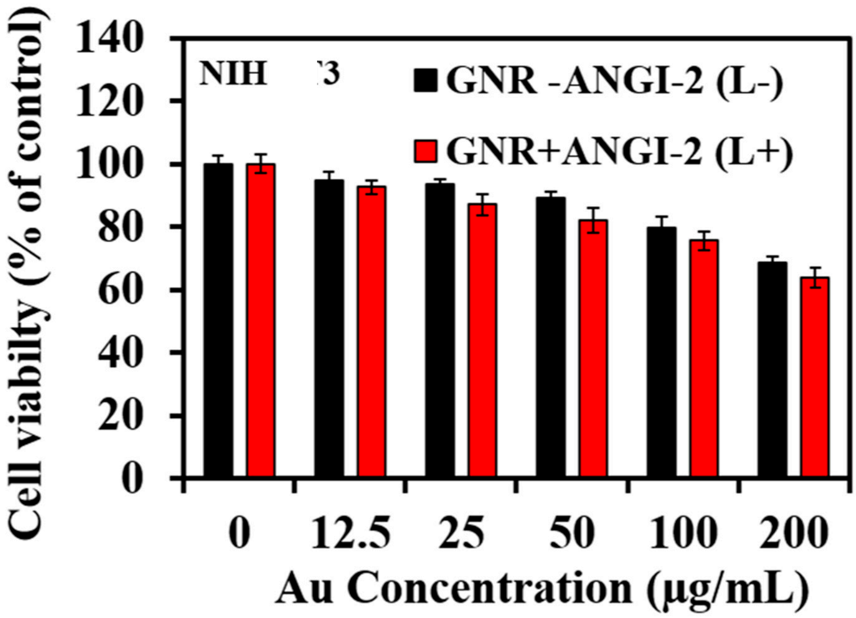

Figure S2. Evaluation of cell viability in NIH3T3 cells.

Percentage of cell viability with GNR-ANGI-2 (L-) and GNR-ANGI-2 (L+) Data were represented as means ± SD.

Western whole blot:

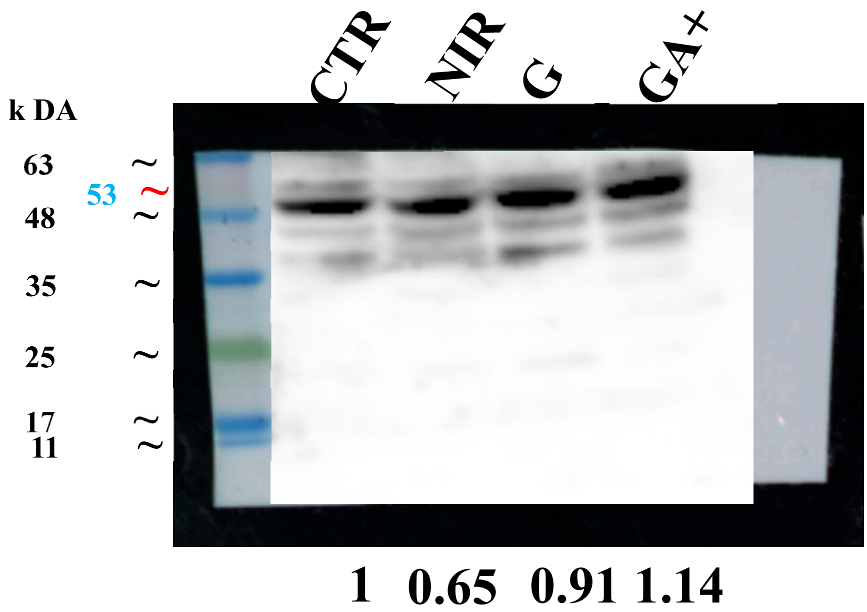

Figure S3. Whole blot of p53

proteintech 10442-1-AP (MW-53 kDa Host-Rabbit, Dilution-1:5000)

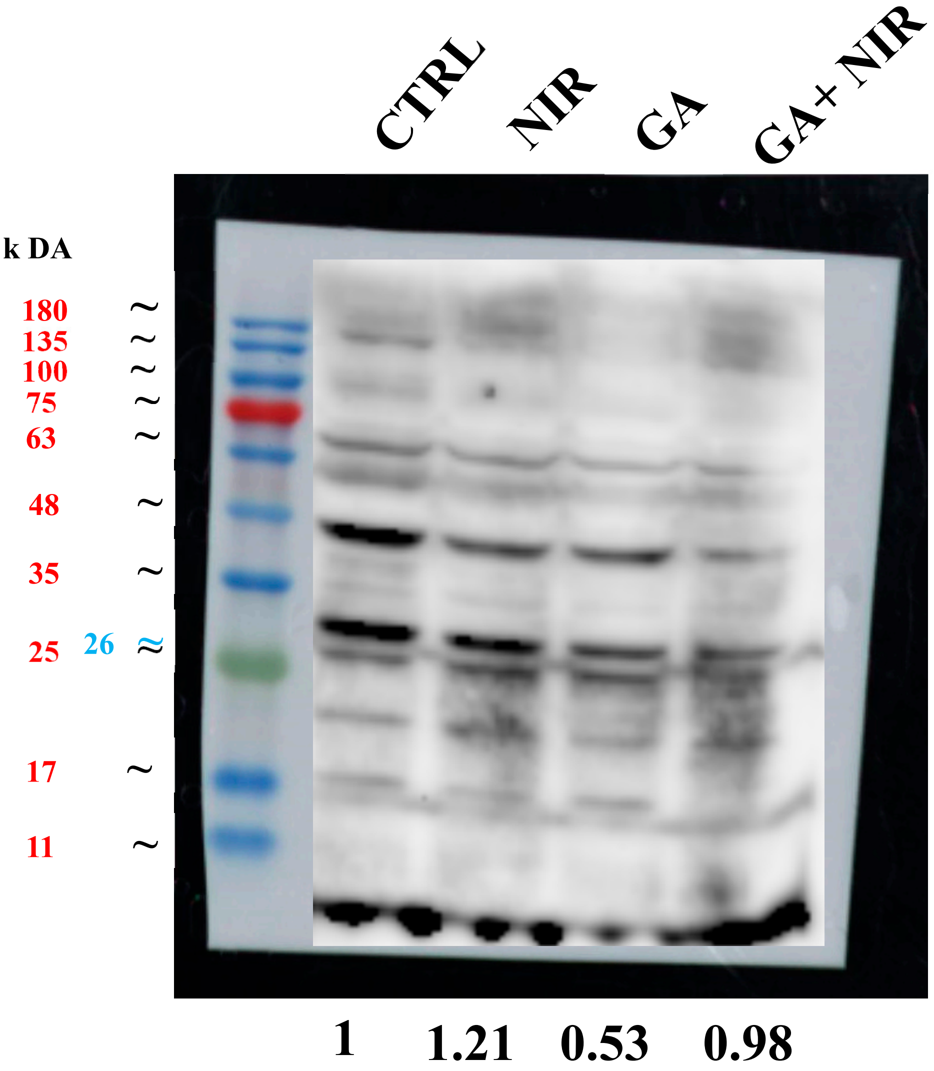

Figure S4. Whole blot of Bcl-2

proteintech 12789-1-AP (MW-26 kDa Host-Rabbit, Dilution-1:1000)

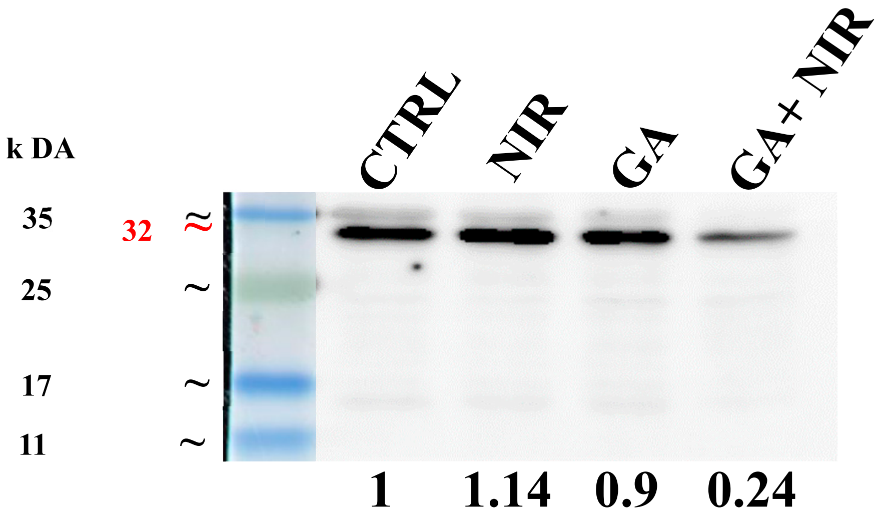

Figure S5. Whole blot of Caspase-3

Novus biotech NB100-56708 (MW-32,17,12 KDa Host-Mouse, Dilution-1:1000)

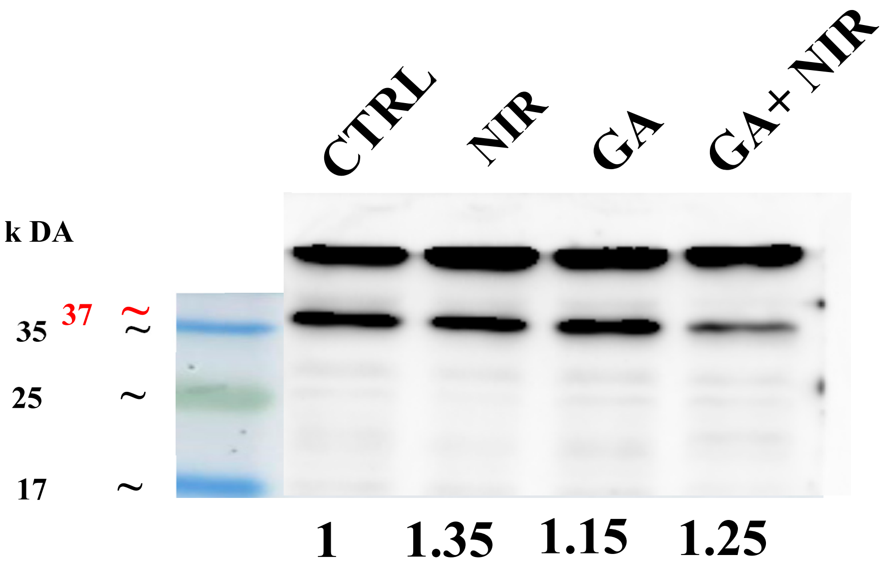

Figure S6. Whole blot of Caspase-7

biotech NB100-56529 (MW-37,20 KDa Host-Mouse, Dilution-1:1000)

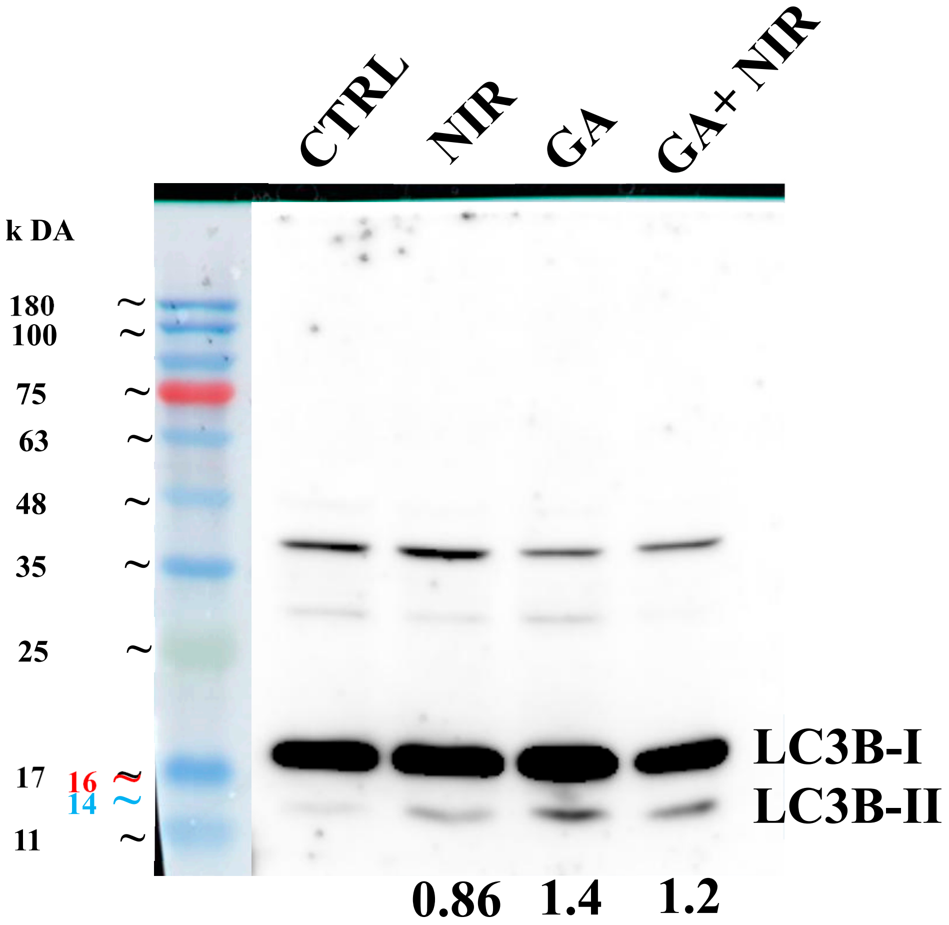

Figure S7. Whole blot of LC3B-I & II

Cell signaling - 2775s (MW-16,14 kDa Host-Rabbit Dil-1:1000)

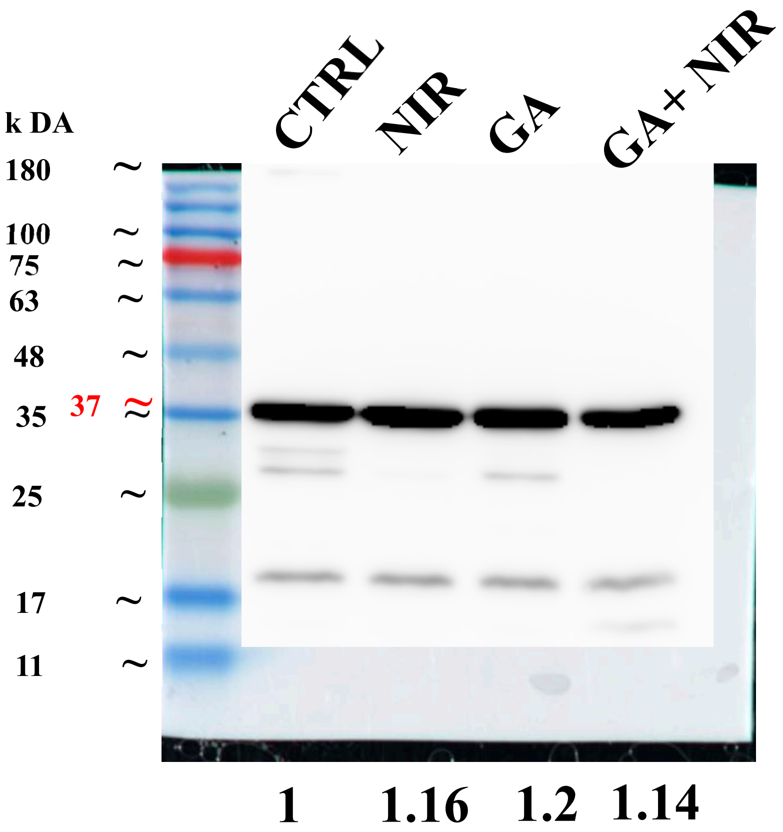

Figure S8. Whole blot of GAPDH

affinity biotech AF-7021 (MW-37 KDa Host-Rabbit, Dilution-1:10,000)

Reference

[1] Sankari, S.S.; Dahms, H.-U.; Tsai, M.-F.; Lo, Y.-L.; Wang, L.-F. Comparative study of an antimicrobial peptide and a neuropeptide conjugated with gold nanorods for the targeted photothermal killing of bacteria. *Colloids Surf. B Biointerfaces* **2021**, *208*, 112117.

[2] Almada, M.; Leal-Martinez, B.H.; Hassan, N.; Kogan, M.J.; Burboa, M.G.; Topete, A.; Valdez, M.A.; Juarez, J. Photothermal conversion efficiency and cytotoxic effect of gold nanorods stabilized with chitosan, alginate and poly(vinyl alcohol). *Mat. Sci. Eng. C-Mater.* **2017**, *77*, 583–593.
